# Interdisciplinary collaboration in the treatment of alcohol use disorders in a general hospital department: a mixed-method study

**DOI:** 10.1186/s13011-022-00486-y

**Published:** 2022-08-12

**Authors:** Nathalie Kools, Guus G. Dekker, Brenda A. P. Kaijen, Bert R. Meijboom, Rob H. L. M. Bovens, Andrea D. Rozema

**Affiliations:** 1 Department of Tranzo, Tilburg School of Social and Behavioral Sciences, Tilburg University, P.O. Box 90153, 5000 LE Tilburg, the Netherlands; 2 Department of Management, Tilburg School of Economics and Management, Tilburg University, P.O. Box 90153, 5000 LE Tilburg, The Netherlands

**Keywords:** Alcoholism, Hospital care, Interdisciplinary care, Interprofessional care

## Abstract

**Background:**

Interdisciplinary collaborations (i.e., where various disciplines work coordinated and interdependently toward shared goals) are stated to yield higher team effectiveness than multidisciplinary approaches (i.e., where various disciplines work in parallel within their professional boundaries) in somatic health care settings. Nevertheless, research is lacking on interdisciplinary approaches for alcohol use disorder (AUD) treatment of hospitalized patients as these types of approaches are still uncommon. This study aims to evaluate an innovative interdisciplinary AUD treatment initiative at a general hospital department by 1) identifying which and to what extent network partners are involved and 2) to explore how network partners experienced the interdisciplinary collaboration.

**Methods:**

A mixed-method study was conducted, using 1) measures of contact frequency and closeness in a social network analysis and 2) semi-structured interviews, which were analyzed thematically. Respondents were network partners of an interdisciplinary collaboration in a general hospital department, initially recruited by the collaborations’ project leader.

**Results:**

The social network analysis identified 16 network partners, including a ‘core’ network with five central network partners from both inside and outside the hospital. The project leader played an important central role in the network and the resident gastroenterologist seemed to have a vulnerable connection within the network. Closeness between network partners was experienced regardless of frequency of contact, although this was especially true for the ‘core’ group that (almost) always consisted of the same network partners that were present at biweekly meetings. Interview data showed that presence of the ‘core’ network partners was reported crucial for an efficient collaboration. Respondents desired knowledge about the collaborations’ effectiveness, and one structured protocol with working procedures, division of responsibilities and agreements on information sharing and feedback.

**Conclusions:**

The design of this interdisciplinary collaboration has potential in improving the treatment of hospital patients with AUD and was evaluated positively by the involved network partners. Interdisciplinary collaborations may offer a critical solution to increase treatment rates of patients with AUD and should be adopted in hospitals on a larger scale. Research towards the effectiveness of interdisciplinary collaborations in the treatment of hospitalized patients with AUD is needed.

## Background

Alcohol use is one of the leading risk factors for disease and injury conditions in almost all European countries [1, 2]. In the past years, (inter)national policies and measures have been adopted with aims to reduce harm due to alcohol use [3]. However, prevalence of alcohol use disorder (AUD) within the European Region remains to be the highest worldwide, with 8.8% of people aged 15 years or older having AUD [3, 4].

Despite the high prevalence and negative impact of AUD, treatment rates among individuals with AUD in Europe are estimated to be only 10% [5–8]. On the one hand, low treatment rates can be explained by patients themselves not seeking treatment, for example due to a lack of problem awareness, stigma or shame, financial barriers or a lack of knowledge who to ask for help [9]. On the other hand, the environment of patients with AUD may fail to detect and/or intervene with the alcohol problems, including relatives, employers, but also patients’ health care professionals. For health care professionals, this might be due to a lack of knowledge and skills, but also because of a lack of protocols on how to deal with patients suffering from AUD [10].

To close this care gap, stronger efforts should be made to find and offer treatment to those with AUD. One place that may be particularly appropriate for doing this is the general hospital setting, asAUD are more prevalent among patients in general hospitals compared to the general population, with an 11.0 to 26.0% prevalence rate [11–16]. In addition to routinely screening alcohol use, additional interventions should be implemented to offer suitable treatment to patients with AUD.

Within somatic care settings like hospitals, multidisciplinary collaborations are frequently recommended for the treatment of patients with more severe AUD or AUD with concurrent medical or mental health problems [17–21]. In multidisciplinary collaborations, professionals operate within their own professional boundaries, each delivering care in parallel according to their own, discipline-specific view [22, 23]. Research on multidisciplinary collaborations for the treatment of hospital patients with AUD is however limited, and a rare example includes the Alcohol Care Teams (ACTs) introduced to hospitals in the United Kingdom [24].

ACTs are clinician-led multidisciplinary teams, consisting of 7-day alcohol specialist nurse services, addiction and liaison psychiatry services, alcohol assertive outreach teams, consultant herpetologists and gastroenterologists with liver disease expertise, that offer customized care for patients with AUD that are hospitalized for any reason [24]. These ACTs seem to reduce acute hospital admissions, readmissions and mortality and improve the quality and efficiency of alcohol care [25, 26]. Although these types of multidisciplinary collaboration approaches offer multiple perspectives, it has been argued that the narrow, discipline-specific views of multidisciplinary collaborations may impede a complete view of the variables associated with complex patients [23].

An interdisciplinary collaboration approach for hospital patients with AUD may therefore be even more effective for the treatment of patients with more severe AUD or AUD with concurrent medical or mental health problems. Professionals in multidisciplinary collaborations work in parallel within their disciplinary boundaries, have clear role definitions, little communication among team members and hierarchical authority levels. In contrast, professionals in interdisciplinary collaborations work coordinated, integrated and interdependently, shared goals and responsibilities, are usually on equal hierarchical levels, have regular meetings to collaboratively discuss, set and carry out treatment plans and show high communication levels among team members [22, 27–29]. This therefore requires more frequent interactions, coordination and commitment of several health care professionals, often from both inside (i.e., doctors, nurses and psychiatrists) and outside the hospital (i.e., general practitioners, social workers and addiction care workers). These characteristics of interdisciplinary collaborations have also been reported in the ‘structuration model of collaboration’ which can be used to analyse complex interprofessional collaborations through four theoretical domains (i.e., ‘shared goals and vision’, ‘internalization’, ‘governance’ and ‘formalization’) [30]. Finally, previous research that compared multidisciplinary with interdisciplinary team approaches found that the latter was associated with higher teamwork and team effectiveness and was recommended in somatic indication fields [28].

Recently, an innovative treatment initiative for patients with AUD was implemented at a gastroenterology and hepatology department of a Dutch general hospital. This department started organizing meetings with several health care professionals from inside and outside their hospital twice a week to offer customized, integrated care to patients that have or are at risk for developing severe AUD (i.e., *Alcohol Use Disorders Identification Test* score of ≥16). Through these meetings, patient support both inside and outside the hospital setting and referral to treatment may be facilitated. This innovative treatment initiative seems to include elements of interdisciplinary collaborations. In the present article, we will refer to this collaboration as ‘the interdisciplinary collaboration’.

To our knowledge, no studies have been conducted on the structure and/or implementation of interdisciplinary approaches for the treatment of hospital patients with AUD. This lack of research on interdisciplinary collaborations in the context of AUD treatment of hospital patients makes it difficult to establish what would constitute ‘best’ practice. More insight is needed in how to best structure and implement interdisciplinary collaboration initiatives. Identifying critical stakeholders and evaluating practical issues in this collaboration could provide important starting points for the implementation of such initiatives within hospitals and ultimately improve treatment rates among individuals with AUD.

This study aims to 1) identify which and to what extent network partners are involved in the interdisciplinary collaboration by performing a social network analysis and 2) to explore how network partners experienced the interdisciplinary collaboration by conducting semi-structured interviews.

## Methods

### Design

A mixed-method study was performed to be able to map a comprehensive account of the interdisciplinary collaboration. In stage one, a social network analysis was performed using an online survey to identify all network partners and to measure 1) frequency of contact and 2) degree of collaboration. This method was chosen, because it allows visualization and analysis of the complex network and interactions between involved partners, and offers opportunities to theorize about relationships between different network partners [31, 32]. Furthermore, it has been argued that social network analysis would be valuable in health care research, even though it has been seldom used in this field [33]. Subsequently, in stage two, semi-structured interviews were conducted to gain in-depth information about how network partners experienced the interdisciplinary collaboration.

### Setting and respondents

This study was carried out at a gastroenterology and hepatology department of a top-clinical hospital in the southern part of the Netherlands. In 2017, this department had 246 admissions, including 175 unique patients (1.4 admissions per patient) and an average length of stay of 6.8 days (*range* 0.5–116 days). Of these patients, 6% had acute pancreatitis due to alcohol and 5% had alcoholic liver cirrhosis. No specific alcohol-related codes were recorded for the remaining patients.

To give the most accurate representation of the collaboration, network partners themselves were included as study population, as they are the core of the interdisciplinary collaboration. First, initial recruitment took place via the collaborations’ project leader, since she had a good overview of the involved network partners. These network partners were invited for the online survey. Second, at the end of the online survey, all respondents were asked if they had contact with any other parties related to AUD treatment, apart from the parties already mentioned. Due to COVID-19, many health care professionals were difficult to reach. Although including all possible network partners at stage one would have been optimal, non-responses was not perceived as problematic since missing parties would be mentioned by other participating respondents. Third, based on the identified network partners from stage one (including suggested other parties), additional recruitment took place for the semi-structured interviews in stage two. It was made sure that the ‘core’ network partners of ‘core’ organizations that were identified in the social network analysis were included in the semi-structured. Respondents interviewed in stage two were not necessarily the same respondents as in stage one, because of a lack of time, staff turnover or some network partners assigned another colleague to participate in the interview.

Inclusion criteria included being a network partner involved in the interdisciplinary collaboration, either by participating in the meetings (in person or by telephone) or by being contacted for consultation, advice or referral for treatment for a patient in the meetings.

### Measures

In both study parts, respondents were first asked socio-demographical questions (i.e., gender, age, education level, work organization, function and years working in organization and function). In stage one, respondents were additionally asked: *“Have you had contact with any (other) party/discipline as a result of a patient in the meeting for alcohol problems?*”, and if so: “*What is the name of this organization and the role of this person?*” Subsequently, frequency of contact and closeness of collaboration between indicated parties was measured using measures of tie strength of Hansen [34] by asking two questions for every party respondents reported: 1) *How frequently do you interact with [insert party] in response to a patient in the meeting for alcohol problems?* and 2) *How close is the collaboration between you and this party with regard to the meeting for alcohol problems?* Respondents answered on 7-point-Likert scales, ranging from 1 (being most frequent or close) to 7 (being least frequent and distant). These questions were repeated until respondents could no longer name a new party.

In stage two, qualitative semi-structured interviews were conducted to explore how network partners experienced the interdisciplinary collaboration. The ‘structuration model of collaboration’ captures successful collaboration in four theoretical domains (i.e., shared goals and vision, internalization, formalization and governance) that are operationalized by ten indicators [30]. This model was chosen as the basis for the interview guide, as it has been shown useful to analyse complex and heterogenous multi-level systems of collaborations in health care settings [30, 35]. Moreover, the model specifically focuses on interprofessional collaboration, which suited best to the objectives of the present study’s interdisciplinary collaboration. Examples of interview questions were: *‘To what extent do you feel free to contribute and express your opinion in the collaboration, and how does this affect the collaboration?* and *‘To what extent are there still missing relationships that are good for cooperation for the purpose of providing good care to the patient?’*

### Procedure

Ethical approval was granted by the Ethics Review Board of Tilburg University (RP52) and all procedures performed were in accordance with the ethical standards of the institutional research. All respondents provided informed consent. Data was collected in Dutch language in both study stages. In stage one (March–June 2020), professionals were contacted by e-mail with an information letter and the request to complete the online survey. Three reminders were sent (i.e., 7 days, 13 days and 19 days after the initial invitation). All participants filled out the survey between 23 April and 7 May 2020. Data was collected through survey software Qualtrics.

In stage two (September–December 2020), professionals were contacted by e-mail with information about the study and an invitation for an interview. When necessary, they received a reminder by e-mail. All interviews were conducted by telephone. Data retrieved from telephone interviews appear to have similar quality to face-to-face interviews [36]. All respondents were interviewed between November and December 2020 by one researcher (BK), a female master’s student with basic interview experience that conducted interviews in the context of her master’s thesis. No field notes were made. Interviews lasted on average 48 minutes (*SD* = 15; *range* 27–74) and were audio-recorded.

### Analysis

**Fig. 1:**
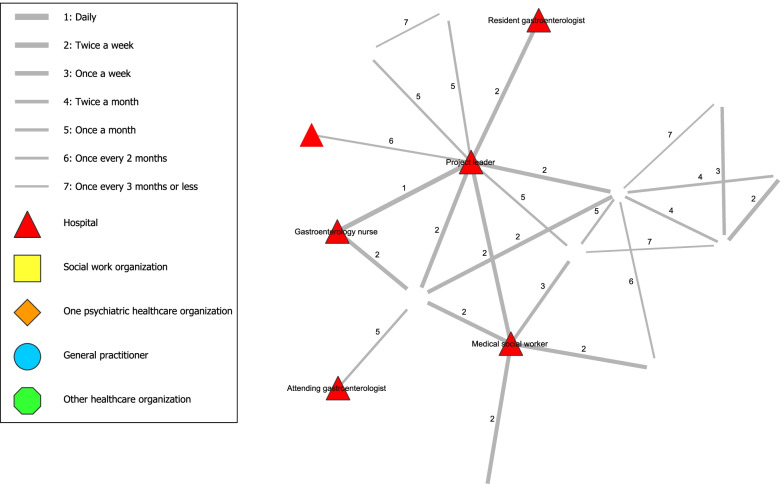
Frequency of contact between network partners of interdisciplinary collaboration

**Fig. 2:**
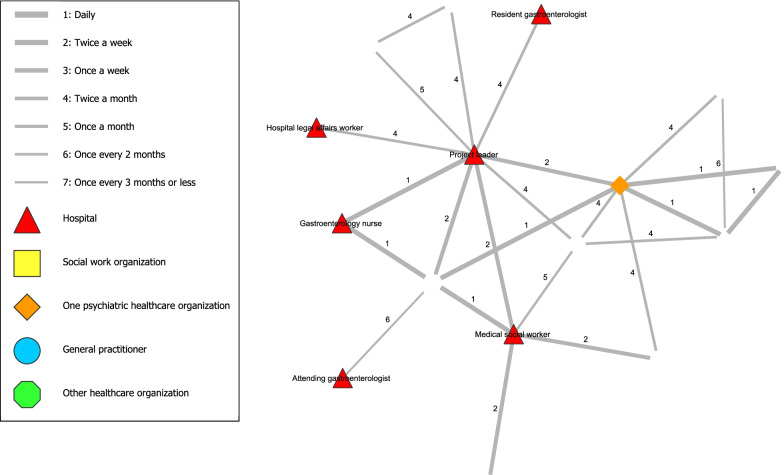
Degree of collaboration between network partners of interdisciplinary collaboration

In stage one, analysis of the Social Network Analysis indicators was conducted using the visualization software Visone 2.18 [37]. An adjacency matrix was created by exporting the data from Qualtrics to Visone, from which figures were created including all the ties between participants involved in the network (i.e., frequency of contact) and the closeness of the ties between them (i.e., degree of collaboration). Two figures were created that showed the total network. The coloured points in the figure represented the network partner of a certain organization and the lines represented the frequency of contact (Fig. 1) or degree of collaboration (Fig. 2).

In stage two, all audiotaped interviews were transcribed verbatim to enable inductive and deductive thematic analysis [38]. In accordance with an essentialist and semantic approach, the experiences, meanings and reality of participants were reported and codes reflected the explicit content of the data [36, 39]. The ‘structuration model of collaboration’ was used to structure and categorize the inductively created codes and themes. This model consists of four theoretical domains, which are operationalized by ten theoretical indicators: 1) domain ‘shared goals and vision’ with indicators ‘goals’ and ‘client-centered orientation vs other alliances’; 2) domain ‘internalization’ with indicators ‘mutual acquaintanceship’ and ‘trust’; 3) domain ‘governance’ with indicators ‘centrality’, ‘leadership’, ‘support for innovation’ and ‘connectivity’; and 4) domain ‘formalization’ with indicators ‘formalization tools’ and ‘information exchange’ [30].

First, two researchers (BK and NK) independently coded the first three transcripts using the software package ATLAS-Ti 8 [40]. Codes were created inductively by the method of constant comparison [38]. Simultaneously, these inductive codes were categorized into one of ten theoretical indicators of the ‘structuration model of collaboration’ (and therefore also in one of the theoretical domains) [30]. Second, codes were then discussed among the two researchers to reflect on interpretations and categorization decisions, and inconsistencies were discussed until consensus was reached. Third, the remaining transcripts were coded by one researcher (BK), by clustering the codes and defining emergent themes, but after every two or three transcripts a consultation took place in which another researcher (NK) reviewed the coding process, after which ambiguities or disagreements were discussed and modified where necessary. Fourth, the final inductive code list, inductive themes and categorization into the theoretical indicators and theoretical domains were checked by the other researchers (ADR and BM) and discussed until consensus was reached. Transcripts were not returned to respondents for comment or correction, nor did respondents provide feedback on the findings.

## Results

The results section is divided into three sections: 1) respondent characteristics, 2) the identification of which and to what extent network partners are involved in the interdisciplinary collaboration (social network analysis) and 3) the exploration of how network partners experienced the interdisciplinary collaboration (evaluation).

### Respondent characteristics

In stage one, twelve professionals were contacted via e-mail, of which eight responded to the survey (response rate 66.7%). Among the eight respondents, six were female (75.0%) and the average age was 46.5 years (*SD* = 11.2). The average years in organization was 17.1 years (*SD* = 12.5) and average years in function was 13.6 (*SD* = 8.1).

In stage two, seventeen professionals were contacted, of which ten participated in the study. Among the ten professionals interviewed, 60% were female, the average age was 38.1 years (*SD* = 11.4) and the average years in function was 7.8 years (*SD* = 7.0). Other respondent characteristics are shown in Table 1.

**Table 1:** Respondent characteristics

Role	Years in function	Organization	From inside or outside hospital	Involved in stage 1	Involved in stage 2
Project leader	4	General Hospital	Inside	X	X
Gastroenterology nurse	8	General Hospital	Inside	X	X
Coordinating gastroenterology nurse	1.5	General Hospital	Inside		X
Resident gastroenterologist	3	General Hospital	Inside		X
Medical social worker	28	General Hospital	Inside	X	
Medical social worker	2	General Hospital	Inside		X
Hospital child abuse officer	11	General Hospital	Inside	X	
Psychiatric nurse specialist	10	Psychiatric health care organization	Inside	X	
Social psychiatric nurse	15	Psychiatric health care organization	Inside	X	X
Psychiatry resident	0.25	Psychiatric health care organization	Inside		X
Community social worker	21	Social work organization	Outside	X	X
Confidential doctor safe at home^a^	3	Safe at home organization^a^	Outside	X	
Addiction specialist	1	Addiction care organization	Outside		X

^a^Safe at home organizations (Dutch: Veilig Thuis) offers advice and support regarding domestic violence and child abuse

### Identification and involvement of network partners

In total, 16 different network partners were identified, which could be divided into five types of organizations (i.e., hospital, social work organization, psychiatric health care organization, other health care organizations and general practitioners).

Figure 1 shows the network related to the interdisciplinary collaboration along with ties representing the existence of a relationship along with the *frequency of contact* between network partners. Five network partners were identified in the centre of the network, including the project leader of the collaboration, social psychiatric nurse, gastroenterology nurse, medical social worker and social worker. These network partners had the most ties to other partners and had relatively high frequencies of contact with multiple partners. In particular, the project leader had ties with almost every partner in the network and worked frequently and closely with most of them. In addition to the ‘core’ group of network partners, a subgroup around the social psychiatric nurse could be identified, consisting of other psychiatric nurses, a psychiatrist and general practitioners. As general practitioners vary from patient to patient, this network partner referred to several different general practitioners.

Moreover, various network partners were identified with ties further away from the centre. These partners generally had lower frequencies of contact and did not have unique ties to other third parties. For example, some network partners were only contacted through a single network partner or organization (i.e., attending gastroenterologist, resident gastroenterologist, hospital legal affairs worker and rehabilitation centre worker). Furthermore, the resident gastroenterologist seemed to have a vulnerable connection within the network, since the resident gastroenterologist generally did attend the biweekly meetings but showed no ties to any other network partners except the project leader in this social network analysis.

Figure 2 shows the network related to the interdisciplinary collaboration along with ties representing the existence of a relationship along with the *degree of collaboration* between network partners. No tie in the Fig. 2 had a closeness of 7 (i.e., distant) and most ties even had a closeness of 4 or closer. In contrast, Fig. 1 contained various values of 7 (i.e., lowest frequency of contact). Therefore, contact between network partners was experienced as ‘close’ regardless of frequency of contact, as even infrequent contacts were perceived as relatively ‘close’.

### Experiences regarding the interdisciplinary collaboration

The experiences regarding the interdisciplinary collaboration are divided into the four theoretical domains of the ‘structuration model of collaboration’ and subdivided into their operationalized indicators: 1) domain ‘shared goals and vision’ with indicators ‘goals’ and ‘client-centered orientation vs other alliances’; 2) domain ‘internalization’ with indicators ‘mutual acquaintanceship’ and ‘trust’; 3) domain ‘governance’ with indicators ‘centrality’, ‘leadership’, ‘support for innovation’ and ‘connectivity’; and 4) domain ‘formalization’ with indicators ‘formalization tools’ and ‘information exchange’ [30]. In total, twenty inductive themes were identified and assigned to these theoretical domains and indicators, as presented in Table 2.

**Table 2:** Coding framework: theoretical domains and indicators, and inductive themes and codes

Theoretical domain	Theoretical indicator	Inductive theme	Inductive code
Shared goals and vision	Goals	1. Providing appropriate care	1.1. Creating appropriate plan for patient
			1.2. Joint division of care responsibilities
			1.3. Incorporating psychosocial aspect into care
			1.4. Arranging post-discharge care
		2. Providing efficient care	2.1. Efficiency and speed in decision making
		3. Prevent alcohol problems	3.1. Early detection and prevention of alcohol problems
	Client-centered orientation vs other allegiances	1. Client-centered interests	1.1. Providing good care
			1.2. Providing preventive care
		2. Professional-centered interests	2.1. Learning (from each other)
			2.2. Working more efficiently
			2.3. Showing one’s own expertise
		3. Hospital-centered interests	3.1 Avoiding unnecessary high bed occupancy
Internalization	Mutual acquaintanceship	1. (Not) knowing each other personally	1.1. By presence or absence at/around biweekly meetings
			1.2. By undertaking joint activities
			1.3. Being able to find each other well and quickly
			1.4. Forgetting when and which discipline to involve
			1.5. Doctors less accessible by hierarchy
			1.6. Due to high staff turnover of resident doctors
	Trust	1. Division of roles	1.1. Clear division of roles among involved disciplines
			1.2. Role uncertainty among inexperienced members
		2. Competences	2.1. Confidence in competences regarding alcohol problems within own department
			2.2. Less confidence in competences regarding alcohol problems of other departments
			2.3. Need for (repeated) training for involved disciplines
			2.4. Desire to increase awareness/insight into importance of alcohol theme in other departments
		3. Collaboration culture	3.1. Pleasant open atmosphere
			3.2. Feeling of connectedness
			3.3. Equality between network partners
			3.4. Disagreement between network partners
		4. Commitment	4.1. Good engagement/commitment among network partners
			4.2. Low commitment of doctors in collaboration
Governance	Centrality	1. Steering	1.1. Project leader as initiator
	Leadership	1. Role of chairperson	1.1 Importance of project leader with guiding role of chairperson during meetings
			1.2. Shared leadership possible
	Support for innovation	1. Project expansion activities	1.1. Initiatives for expansion to other hospital departments
			1.2. Additional activities around network promotion
		2. Need for research	2.1. Lack and need for insight into actual effect of interdisciplinary collaboration
	Connectivity	1. Contact moments design	1.1. Fixed biweekly meetings
			1.2. Preferring physical meetings over digital
			1.3. Desiring possibilities to start actions earlier than biweekly meetings
			1.4. Limited accessibility to collaboration for other departments
			1.5. Fewer contact moments and poorer collaboration due to COVID-19
			1.6. Waiting lists of external partners hinder collaboration
			1.7. Importance of reminding each other of biweekly meetings
		2. Composition of network	2.1. Presence of fixed core network partners
			2.2. Importance of involvement/presence of different and fixed expertises
			2.3. Changes in composition of network partners is logical
			2.4. Desire to involve various external parties more/earlier
			2.5. Low involvement of various external parties is difficult
Formalization	Formalization tools	1. Protocols	1.1. Bureaucracy and protocols of hospital hinders cooperation
			1.2. Inclusion of protocol for alcohol problems in induction pack for resident doctors
			1.3. Desire for structured protocol with working procedures and division of responsibilities
			1.4. Protocols not followed
		2. Funding	1.1. Difficulties regarding funding of external partners hinders collaboration
	Information exchange	1. Ways of exchanging information	1.1. During biweekly meetings
			1.2. Via telephone or e-mail
			1.3. Processing information and action items in Electronic Health Records (for hospital professionals)
		2. Evaluations of information exchange	1.1. Lack and need for information about post-discharge care process
			1.2. Gaps in reporting/transmission of information
			1.3. No changes desired

#### Shared goals and vision

The shared goals and vision domain is operationalized by two indicators: 1) goals and 2) client-centered orientation vs other allegiances.

In total, three goals were mentioned: a) providing appropriate care, b) providing efficient care and c) preventing alcohol problems. Overall, respondents found the provision of good and appropriate care for patients pivotal. This entailed extending standard “medical-only” care to psychosocial and post-discharge care, but also providing preventive care for alcohol problems rather than just curative:

*“If we manage to keep that man or woman away from alcohol through the advice of the meetings, yes, then we have also solved pancreatitis for the future.” – R7, Resident gastroenterologist, 3 years in function.*

Moreover, respondents mentioned efficiency in decision making and care provision to be another major goal:

*“Because in the end I think that without the meetings you can achieve the same end result, only that takes a lot of time and a lot of consultation.” – R4, Medical social worker, 2 years in function.*

In addition, three interests (client-centered orientation vs other allegiances) were mentioned by respondents: a) client-centered interests, b) professional-centered interests and c) hospital-centered interests. Client-centered interests were related to providing appropriate, preventive care. In contrast, professional-centered interests included being interested in the collaboration because it gave them opportunities to learn from other disciplines’ expertise or to making their own discipline and expertise more visible to other network partners.

*“And maybe very selfishly, I believe that my specialism should be more to the forefront. So that would be purely my own interest. Of showing what we can do and sharing our knowledge with other specialists.” – R1, Addiction specialist, 1 year in function.*

These professional-centered interests were considered to contribute to better patient care provision eventually. Finally, some hospital-centered interests were mentioned as hindering treatment of patients with AUD, namely trying to avoid unnecessary high bed occupancy:

*“And also very hard practical financial concerns within such a hospital. It’s also about discharge pressure and discharge rate. That’s also a reality.” – R5, Community social worker, 21 years in function.*

#### Internalization

The internalization domain is operationalized by two indicators: 1) mutual acquaintanceship and 2) trust.

As for the mutual acquaintanceship indicator, the only one category of responses concerned was (not) knowing each other personally. Many respondents indicated that they knew each other well, mainly due to presence at the biweekly meetings. Respondents elaborated on this by stating that they were able to find each other well and quickly:

*“Because you are actually always with the same people. Yes, then you have every confidence in them. And then you get more and more connected. So that makes it all easier.” – R9, Coordinating gastroenterology nurse, 1.5 years in function.*

This mutual acquaintanceship was however less evident for network partners only occasionally present at the biweekly meetings or involved indirectly, such as the resident gastroenterologist, addiction specialist and hospital child abuse officer. Moreover, various comments were made regarding doctor-related factors that hinder collaboration, such as infrequent presence at the biweekly meetings, high staff turnover resulting in unawareness of the meetings repeatedly and perceived hierarchy which makes one less likely to approach doctors:

“*They’ve been made aware of that several times. Some of the doctors are always there, but other doctors also say: “Yeah, what am I supposed to do there?” So, some repetition in that might be necessary, because they are also resident doctors who change every time.” – R2, Gastroenterology nurse, 8 years in function.*

For the trust indicator, responses could be divided into four categories: a) division of roles, b) competences, c) collaboration culture and d) commitment. Respondents mentioned they had trust in the division of roles, as it was clear what they could expect from each other:

*“Everyone knows a bit of what to expect from another.” – R2, Gastroenterology nurse, 8 years in function.*

They however did notice some role uncertainty among more inexperienced members:

*“I sometimes find that some disciplines do take a somewhat wait-and-see role, and others within the meetings do take a more directive advisory position. I think that also depends on the person. And I also think that if you do it more often, then you find it easier.’ – R4, Medical social worker, 2 years in function.*

Also, respondents mentioned being positive about the competences of the other network partners. More specifically, due to training in the gastroenterology and hepatology department, alcohol-related knowledge had been improved significantly. Respondents however did mention that these trainings should be repeated every year and ideally should be extended to other hospital departments as alcohol-related knowledge seemed to be lower in other departments.

*“I think everyone has, um, enough knowledge in their own field. And so that complements, well, in a nice way.” – R10, Psychiatry resident, 0.25 years in function.*

Moreover, respondents mentioned that the collaboration culture was pleasant. Respondents experienced equivalence and a sense of belonging in the collaboration, regardless of role or discipline. They also described feeling free to speak openly and ask critical questions. Finally, respondents emphasized that they felt that network partners had a strong commitment to make the collaboration a success. According to respondents, this commitment was demonstrated by the presence of ‘core’ network partners in the collaboration and the immediate acceptance and execution of patient plans formulated in the biweekly meetings.

*“I also notice the commitment of everyone, everyone who sits at the table is sure to be there. I think everyone sees the urgency and everyone wants to work with it.” – R6, Project leader, 4 years in function.*

#### Governance

The governance domain is operationalized by four indicators: 1) centrality, 2) leadership, 3) support for innovation and 4) connectivity.

For the centrality indicator, the only category of answers concerned the steering of the collaboration. Respondents mentioned that the project leader fulfilled a steering, strategic role in the on-going implementation of collaborative processes and structures. For the leadership indicator, the only category of answers concerned the role of the chairperson. Again, respondents mainly spoke about the important role of the project leader by being the chair of the biweekly meetings and by providing direction, clarity, coordination of agreements, efficiency and reminders. Respondents did mention that shared leadership was possible within the collaboration, as other network partners occasionally had to take on the role of chair during COVID-19 times.

*“The project leader gives a lot of guidance on that. And not only in the meetings, but also in the sharing of information, planning, and the training that we did last year for the nurses. Yes, a very big role is reserved for the project leader.” – R5, Community social worker, 21 years in function.*

For the support for innovation indicator, responses could be divided into two categories: a) project expansion activities and b) need for research. For project expansion activities, respondents mentioned that extra activities were organized to further strengthen the network, by for example extending the interdisciplinary collaboration to other hospital departments, organizing a symposium among themselves, participating together in other projects and planning to visit each professional’s organization for a ‘peek behind the scenes’. In contrast, for need for research, respondents mentioned that they currently lacked, and had a need for, insight into the actual effect of the interdisciplinary collaboration. This would include insight into the value and benefit of each involved network partner in the interdisciplinary collaboration. These responses implied that no specific expertise was available to support the collaboration.

*“Well, I think you want to know as a health care provider that it’s useful that you’re there, but also that you know that the meeting also is effective. So do people actually stop drinking alcohol because the meeting is there?” – R7, Resident gastroenterologist, 3 years in function.*

For the connectivity indicator, two types of responses could be distinguished: a) design of contact moments and b) composition of network. For the design of contact moments, respondents mentioned that the biweekly meetings were the main place for discussion between network partners. Although these biweekly meetings were experienced as positive in general, it was noted that it is sometimes inconvenient to have to wait for days of the meetings to start action points, as it may unnecessarily prolong hospitalization.

*“The meetings are scheduled by default. It’s not an ad hoc thing. I think ad hoc within a hospital often tends to get lost.” – R7, Resident gastroenterologist, 3 years in function.*

In addition, as for the composition of the network, respondents described that a fixed ‘core’ group of network partners was usually present at the biweekly meetings. This was perceived as necessary for successful meetings. The presence of a gastroenterologist was considered as crucial for the decisiveness and support for agreements:

*“You immediately notice if someone is not there, for example, from [name of psychiatric nursing organization], then you don’t actually have a meeting. And also medical social work, doctor, nurse, they must all be present for a good meeting.” – R9, Coordinating gastroenterology nurse, 1.5 years in function.*

*“Well, at the end of the day, doctors are the head practitioners, right? So, they’ll have to agree to some things eventually too.” – R8, Social psychiatric nurse, 15 years in function.*

In contrast, it was noted that not all network partners needed to be present at all times, as not all network partners were always relevant to the patient in question, such as addiction care workers, child abuse officers, social workers and general practitioners. Only when relevant, external network partners were invited for the meetings or contacted by e-mail or phone to seek advice or for further coordination. Therefore, and also due to alternating shift schedules, changes in the compositions of network partners present in each meeting was mentioned as logical and inevitable.

#### Formalization

The formalization domain is operationalized by two indicators: 1) formalization tools and 2) information exchange.

For the formalization tools indicator, responses could be divided into two categories: a) protocols and b) funding. Respondents mentioned that despite the existence of a protocol that was developed during the introduction of the interdisciplinary collaboration, there was still a need for a more structured protocol regarding working procedures and division of responsibilities:

*“Then everyone can also find it. Then it’s also just clear who can be involved for what at what point in the treatment.” – R10, Psychiatry resident, 0.25 years in function.*

Moreover, respondents mentioned that protocols were not always followed, as for example general practitioners were not always contacted and some partners were not always present at the biweekly meetings. As for funding, respondents mentioned some difficulties regarding the funding of external parties, which hindered including external parties in the collaboration, in particular addiction care parties:

*“Currently it’s not regulated that certain care is paid for, so you can offer that very little as well. Otherwise, my manager gets mad at me like: “Hey, you’re not bringing in any money.” – R1, Addiction specialist, 1 year in function.*

For the information exchange indicator, responses could be divided into two categories: a) ways of exchanging information and b) evaluations of information exchange. As for the ways of exchanging information, respondents described that all hospital professionals could access and store information in the Electronic Health Record through a special “meeting alcohol problems”-template. External network partners were typically briefed via e-mail or phone. In addition, all necessary information was presented during the biweekly meetings.

*“In the meetings we actually also have a function to collect what is there so far. And we look at what still needs to be done in terms of information. Who do we still want to call or approach or make contact with?” – R4, Medical social worker, 2 years in function.*

However, as for evaluations of information exchange, respondents did indicate a lack of information about the post-discharge process. Although active feedback was not perceived as crucial, respondents mentioned it would be desirable to have the opportunity to trace back the course of a post-discharge process.

*“I think that feedback is desirable from time to time, so that you can respond to the fact that a certain situation went well or that a certain situation or plan didn’t turn out the way we thought it would.” – R2, Gastroenterology nurse, 8 years in function.*

Moreover, various issues were mentioned as hindering collaboration, including rigid processes and protocols due to the hospital’s bureaucracy*.*

## Discussion

### Key findings

The design of this collaboration has potential in improving the treatment of hospital patients with AUD and was evaluated positively by the involved network partners. The social network analysis showed that the project leader played an important central role in the network. Five central network partners, including the project leader, formed the ‘core’ of the network. The resident gastroenterologist seemed to have a vulnerable connection within the network, although being responsible for a significant portion of the patient’s care. Contact between network partners was experienced as ‘close’ regardless of frequency of contact, as even infrequent contacts were perceived as relatively ‘close’.

The semi-structured interviews demonstrated that all partners shared the same goal: providing appropriate care for patients. Furthermore, respondents described a strong mutual acquaintanceship and trust among the ‘core’ group of network partners, partly because these network partners routinely attended the biweekly meetings. Additional network partners were only invited when relevant and useful to specific patient cases, but where therefore less mutual acquainted. The project leader’s role was perceived as important, although shared leadership was possible. Finally, respondents expressed a desire for knowledge about the collaborations’ effectiveness and a more structured protocol with working procedures, division of responsibilities and agreements on information sharing and feedback.

Consistent patterns of data are shown when combining both study methods. First, the importance of the project leader is shown by the central position in the social network analysis and statements on the importance of the project leader (e.g. by guiding and structuring the collaboration). Second, the vulnerable link of the resident gastroenterologist is demonstrated in both study methods, following the single tie from project leader to this partner in the social network analysis and mentions of hindering doctor-related factors (e.g. infrequent presence at meetings, high staff turnover and perceived hierarchy). Third, the social network analysis showed a ‘core’ group of network partners and that closeness was experienced regardless of frequency of contact. This was confirmed by feeling connected to each other. However, this was especially true for the ‘core’ group that were reported to (almost) always being the same network partners. Less mutual acquaintanceship was reported for network partners only occasionally present at the biweekly meetings or involved directly. Nevertheless, according to network partners, presence of the ‘core’ network partners was crucial for having an efficient collaboration.

### Interpretations of the key findings

When comparing the present collaboration to the multidisciplinary ACTs in the United Kingdom, we see that shared leadership was reported to be present in the present interdisciplinary collaboration, which is not the case in the ACTs, reflecting one of the differences between multi- and interdisciplinary collaborations [24, 28]. Previous research shows a higher team effectiveness in interdisciplinary (i.e., with shared leadership) in comparison to multidisciplinary collaborations. Therefore, multidisciplinary ACTs may achieve an even higher effectivity when incorporating elements of interdisciplinary collaborations such as shared leadership. In addition to studying the effectiveness of interdisciplinary collaborations in general, which was an expressed need of respondents, future research should also investigate the (additional) effectiveness of shared leadership in AUD treatment of hospital patients [28].

Furthermore, it seems that involvement of several core partners from different organizations are crucial to successful collaboration. However, it is not obvious that all partners work together. For example, whereas ACTs stated the importance of close working relationships with regional addiction psychiatrists, in our current study the addiction care partner seems less involved [24]. Conversely, the social worker (i.e., social work organization) is greatly involved in the present study, which is not always obvious, when comparing it to other integrated hospital care for childhood overweight where social workers were less involved [41]. Despite its acknowledged importance, actively involving all relevant network partners seems difficult, due to for example lack of intrinsic motivation of a network partner, financial barriers or not knowing network partners [10, 41]. It is therefore recommended to actively monitor involvement of network partners in these types of collaborations. Another finding that requires attention is that the resident gastroenterologist only has a link to the project leader, which might be explained by the high staff turnover due to short rotations of junior doctors, as these short rotations are known to hinder (the continuity of) interdisciplinary care [42]. Respondents however repeatedly emphasized the importance of presence and involvement of this partner**,** as this partner contributes to a significant portion of the patient’s care. Since continuity of doctors’ involvement is reported to be important for interdisciplinary care [42], efforts should be made, for example by giving training emphasizing the importance of the interdisciplinary collaboration and being present at the network meetings, and induction packs with protocol information [10, 42]. In this way, resident gastroenterologists can get familiar rapidly in the new working environment and, in this case, the interdisciplinary collaboration.

### Limitations and strengths

A limitation of the present study was that almost every patient had a different general practitioner, which made it difficult to reach general practitioners and due to the COVID-19 pandemic even impossible. Data from this partner however would have provided an even more complete picture of the collaboration, since social network analysis and the interviews showed that this partner is important in the collaboration. Nevertheless, the use of mixed-methods in the present study resulted in rich and detailed data of this innovative interdisciplinary collaboration.

## Conclusions

The design of this interdisciplinary collaboration has potential in improving the treatment of hospital patients with AUD and was evaluated positively by the involved network partners. Involved network partners had similar goals, leadership seemed exemplary, and both network analysis and interviews showed that contact was experienced as frequent and close among ‘core’ network partners. Presence of the five ‘core’ network partners at the bi-weekly meetings seemed to be a prerequisite for successful collaboration, with additional network partners invited only based on specific patient characteristics. Further improvements could be made in involving resident physicians more, providing knowledge about the collaborations’ effectiveness and implementing a more structured protocol with working procedures, division of responsibilities, and agreements on information sharing and feedback.

Interdisciplinary collaborations may offer a critical solution to increase treatment rates of patients with AUD and should be adopted in hospitals on a larger scale. Other collaborations in this field should pay special attention to assigning a project leader, implementing regular meetings where all core network partners are present, actively involving all relevant parties and developing a structured protocol. Research towards the effectiveness of interdisciplinary collaborations in the treatment of hospitalized patients with AUD is needed.

## Data Availability

The data generated and analyzed during this study are not publicly available due to ERB restrictions. Non-identifiable data are however available from the authors upon reasonable request and with permission from the ERB of Tilburg University.

## Abbreviations

ACT: Alcohol Care Teams
AUD: Alcohol Use Disorder
ERB: Ethics Review Board
