# Correction: Interdisciplinary collaboration in the treatment of alcohol use disorders in a general hospital department: a mixed-method study

**DOI:** 10.1186/s13011-022-00492-0

**Published:** 2022-09-06

**Authors:** Nathalie Kools, Guus G. Dekker, Brenda A. P. Kaijen, Bert R. Meijboom, Rob H. L. M. Bovens, Andrea D. Rozema

**Affiliations:** 1 Department of Tranzo, Tilburg School of Social and Behavioral Sciences, Tilburg University, P.O. Box 90153, 5000, LE Tilburg, the Netherlands; 2 Department of Management, Tilburg School of Economics and Management, Tilburg University, P.O. Box 90153, 5000, LE Tilburg, The Netherlands

**Correction: Subst Abuse Treat Prev Policy 17, 59 (2022)**

**https://doi.org/10.1186/s13011-022-00486-y**

Following publication of the original article [1], we have been notified that Figs. 1 and 2 were not published correctly. The correct Figs. 1 and 2 should be as per below:

**Fig. 1:**
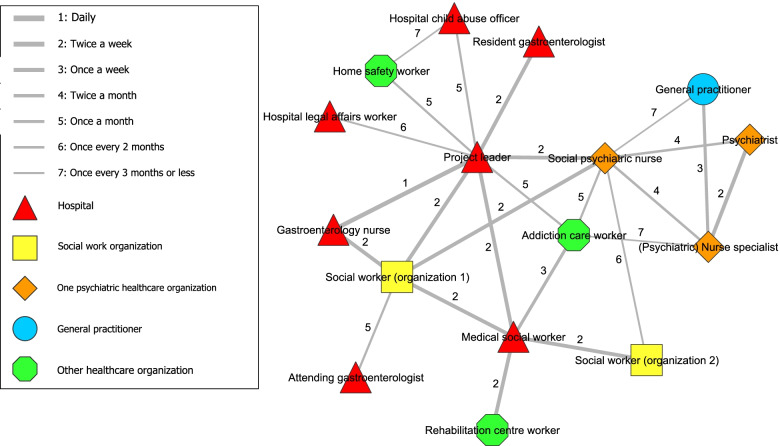
Frequency of contact between network partners of interdisciplinary collaboration

**Fig. 2:**
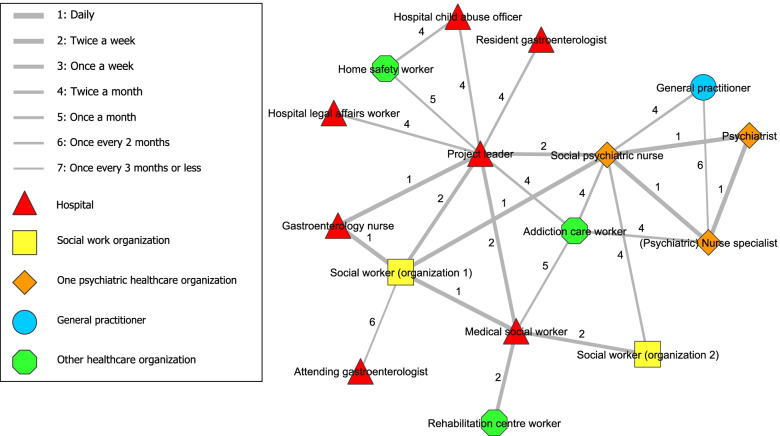
Degree of collaboration between network partners of interdisciplinary collaboration
